# Obstetric Tables: Comprising Graphic Illustrations, with Descriptions and Practical Remarks; Exhibiting on Dissected Plates Many Important Subjects in Midwifery

**Published:** 1848-06

**Authors:** 


*Obstetric Tables: comprising graphic illustrations, with descriptions and practical remarks; exhibiting on dissected plates many important subjects in Midwifery.* By G. Spratt, Surgeon-Accoucheur. First American Edition, from the fourth and greatly improved London edition; carefully revised, and with additional notes and plates. Thomas, Cowperthwait & Co. 1848.

The title of this work, which we have copied at length, describes very accurately its character. The plates, which are somewhat after the manner of Hogben's, are handsomely engraved and coloured, and show very clearly the different positions of the fœtus, and the relations of the presenting parts to the maternal structures, and the practice to be pursued under the various circumstances. The descriptions and directions are brief, explicit, and generally sound—in fact, the doctrines are essentially those of Denman and Smellie. On the whole, we regard the obstetric tables as a very useful publication. Works of this description have a great advantage over simple didactic treatises, in the clearer and more lasting impression which they make upon the student's mind, as is always the case when the understanding is addressed through the eye.

